# The International Ovarian Tumor Analysis-Assessment of Different Neoplasias in the Adnexa (IOTA-ADNEX) Model Assessment for Risk of Ovarian Malignancy in Adnexal Masses

**DOI:** 10.7759/cureus.31194

**Published:** 2022-11-07

**Authors:** Srinidhi Cherukuri, Shubhada Jajoo, Deepika Dewani

**Affiliations:** 1 Obstetrics and Gynaecology, Jawaharlal Nehru Medical College, Datta Meghe Institute of Medical Sciences, Wardha, IND

**Keywords:** prognosis, ultrasound, adnexal masses, ovarian malignancy, iota-adnex model

## Abstract

Ovarian cancers are one of the major leading causes of death across the world. In addition to many challenges to diagnose the disease, it is also hard to predict the type of cancer with effective tools and technology. Many attempts have been made to diagnose ovarian malignancies using ultrasonography, MRI, and CT scans, but seldom will they give the clinician a clear understanding of cancer’s type and stage. It is of utmost importance to understand the mass peri-operatively, which will help the clinicians to decide on the course of management mortality. With technological advancements, many predictive models have come into the picture. Many of those were dependent on the Serum CA-125 markers. With ultrasonography machine usage, the International Ovarian Tumor Analysis (IOTA) group has developed a Simple Rules model, Logistic Regression (LR) models, and, most recently, the IOTA-assessment of different neoplasias in the adnexa (IOTA-ADNEX) model. It has been found to be effective and reliable among all the tools developed in the past. The ADNEX predicts the type of cancer (benign or malignant) and stages of cancer (borderline, Stage I, Stages II-IV, and secondary metastatic). These models can be used for people who are coming with persistent adnexal masses in the ovarian region, para ovarian region, or in the tubes and are recommended for the surgeries. The model is developed by a team of clinicians and statisticians, based on ultrasound and clinical data. This article reviews the IOTA-ADNEX model as a tool for predicting ovarian malignancies in people coming with adnexal masses, especially in comparison with other methods and models. It also tests its effectiveness in the hands of experienced technicians and non-expert technicians.

## Introduction and background

Cancer is known to be caused by mutations due to environmental factors, errors during DNA replication, or may be inherited [1]. Aging is one of the main risk factors for carcinogenesis [2]. Out of 172 countries, 91 countries attribute cancer as the first or second leading cause of death [3] and ranked third or fourth in the other 22 countries [4]. In India, cancer is the second leading cause of death in urban areas and the fourth leading cause of death in rural areas [5]. Autopsy research conducted by India's finest postgraduate medical institute indicated that 25.8% of malignancies were misdiagnosed [6]. In most developing countries, cervical cancer is one of the common causes of death in females [7]. The deaths due to cervical cancer in low- and middle-income countries reveal health inequities. 86% of deaths are in these countries and, hence, death due to cervical cancer is a crucial indicator to assess health inequities [8]. Ovarian tumors are seen frequently by all gynecologists, and prior to any surgical or non-surgical management, a proper diagnosis of these masses prior to the surgery is critical because effective care and management rely on a better understanding of the type of tumor.

Many researchers have tried to develop a comprehensive screening tool, but due to poor performance, they were not streamlined [9]. An accurate diagnosis of ovarian cancer is a challenge for gynecologists as the symptoms appear in a very bizarre and non-specific way [10]. Initially, ovarian cancers can be diagnosed by detecting adnexal masses. Laparotomy or laparoscopy with histopathology gives the best accurate prognosis of the disease [11]. It takes an experienced gynecologic oncology surgeon in a multispecialty cancer or tertiary hospital to perform these kinds of surgeries. There is an increase in survival of the patients when a high risk of malignancy, stage, and type of cancer is known well in advance, especially pre-operatively. Many present themselves in the local or primary health centers with adnexal masses and some non-specific symptoms. It is crucial to decide the course of treatment for such patients, whether to conduct the operation, and decide the type and extent of the operation before the surgery [12-14]. Prior data on the type of tumor (benign or malignant) will help in better management of the patient. With an effective tool for assessing these pelvic masses or adnexal masses, more patients ca get first-line treatment in the early stages, reducing mortality and morbidity. However, there are no established population-based screening tools available for the disease. For such patients, a sensitive, specific, and validated tool for the diagnosis of ovarian cancer is an urgent need [11].

A specific tumor marker called Serum CA-125 is a crucial marker for diagnosing ovarian cancer. Few studies have found that elevation of this marker concentration is found in 85% of epithelial ovarian cancer with a cut-off level of 35 U/ml [14]. Various software programs, scoring systems, and mathematical models depend on this marker. In addition to the Serum CA-125 marker, ultrasonographic findings, CT scans, and triage with MRI helped in accurate distinction of ovarian cancer as benign and malignant [15]. To identify the presence of ovarian mass, gynecologic ultrasonography is the most commonly used tool [16]. Not only identify the presence of any kind of mass but also differentiates the benign and malignant tumor. Furthermore, the International Ovarian Tumor Analysis-assessment of different neoplasias in the adnexa (IOTA-ADNEX) model is used to determine the treatment plan and course of action. Furthermore, the IOTA-ADNEX model is used to determine the treatment plan and course of action, which helps the gynecologist to reduce mortality and morbidity. Another interesting finding is that ovarian malignancies are not common among gynecologic tumors but they are a reason for concern due to their fatality with high chances of recurrence. But early detection through ultrasonography in turn improves the patient’s survival rate thus providing cost-effective treatment and follow-up [17].

Several scoring tools and techniques, such as Risk of Malignancy Index (RMI) and Risk of Ovarian Malignancy Algorithm (ROMA), are designed to identify the adnexal masses and differentiate them into categories of benign and malignant. In 2005, the IOTA group developed many new algorithm-based risk predictive models. Logistic Regressions 1 and 2 (LR1 and LR2) are modified tools made using sonographic characteristics of Simple Rules and LR. The predictive models performed better compared to any previously designed models. In 2014, IOTA designed a new model with better performance, the ADNEX model. It is the first risk model that not only tells us whether the tumor is benign or malignant but also the stage and type of cancer, such as borderline, Stage I, Stages II-IV, and secondary metastatic [17].

## Review

The IOTA-ADNEX model

The ADNEX model can be found on the IOTA website. The model was developed by a team of clinicians and statisticians. It has studied over 6,000 women from 24 centers across 10 different countries. The model is validated externally by IOTA and internally by many other studies. It uses nine predictors, three clinical variables, and six ultrasound variables.

The nine predictors of this model are: age (years), Serum CA-125 level (U/mL), type of center (oncology referral center vs non-oncology center), maximum diameter of the lesion (mm), number of papillary projections (0, 1, 2, 3, or > 3), maximum diameter of the largest solid part of the lesion (mm), more than 10 cyst locules (yes or no), ascites (yes or no), and acoustic shadows (yes or no). The results of all predictors are acquired in both graphic and numerical forms to present the likelihood of malignancy [18]. The present article aimed to provide a review of the efficacy of the IOTA-ADNEX model as a predictive tool for the risk of ovarian malignancy in adnexal masses.

Few studies that have addressed the reliability of this model on the borderline ovarian tumor (BOT) suggested that this is an effective tool to differentiate the BOT from other tumors. One of the unique studies done by Gaurilcikas et al. studied the utilization of the ADNEX model on BOT. They used the web application of the ADNEX model [19]. Different cut-offs, such as 3%, 5%, 10%, and 20%, were tested. In addition, Absolute Risk (AR) and Relative Risk (RR) were applied to the data. The studies by including and excluded the Serum CA-125 marker. They found that AR performed better with smaller cut-off values of 3% and 5%, whereas RR performed better for higher cut-offs. They concluded that a cut-off value of 10% should be utilized for AR and RR for diagnosing the BOT [19]. Another study conducted by Peng XS et al. on evaluating diagnostic values for differentiating benign and malignancy suggested that a universal fixed value cannot be assigned for all the setups [20]. It depends upon the population, settings, clinicians, and local protocols. They also found that this model gives accurate results without using the Serum CA-125 marker [20]. They also found that results via the ADNEX model and those with histopathology were similar, hence proving that this tool is effective and can be considered as best among all other tools. This model can be utilized along with other methods like ultrasonography and try to triage them to make better decisions [21].

Comparison of subjective assessment and IOTA-ADNEX models for ruling out malignancy in adnexal masses

Few studies have revealed that the IOTA-ADNEX model is reliable and gives confidence to gynecologists to determine the type of tumor and to further plan action. One such study was conducted by Soo Young Jeong and colleagues where they compared the IOTA-ADNEX model with the subjective assessment by an expert [16]. Interestingly, they found out that the IOTA-ADNEX model is excellent at identifying ovarian masses and it also has a high specificity in evaluating the risk of malignancy [16]. With accuracy in identifying ovarian tumor features, the ADNEX model also has a strong negative predictive value and exclusion of malignancy. Furthermore, the ADNEX model has effectively distinguished Stages II-IV ovarian cancers from other tumors [22]. Some studies also found that the IOTA-ADNEX model has a polytomous discrimination index (PDI)” of 0.61, whereas random performance would have a PDI of 0.25. In the distinction between benign and malignant adnexal masses, ADNEX was demonstrated to be equally or more accurate than subjective evaluation or the two-step technique [23]. Another research aimed at validating the ADNEX model's usage by Level II examiners found that it can be utilized successfully by inexperienced examiners and sonographers as well [23].

Comparison of RMI and IOTA-ADNEX models

The RMI is another simple and practical index to rule out malignancy from the ovarian mass. “RMI is calculated with a simplified regression equation obtained from the product of menopausal status score (M), ultrasonographic score (U), and an absolute value of serum CA-125” [24]. There are four types of RMI scores: RMI1, RMI2, RMI3, and RMI4. Few studies have found that there is no statistical difference in all four types of RMI scores and yielded more or less the same results [24]. Even though the RMI method is simple, it does not provide any diagnostic advantages. The RMI's limitations include that it lacks in estimating the risk of malignancy and its high dependence and reliance on Serum CA-125. In addition, it has very poor sensitivity for early-stage invasive and borderline illness, particularly in pre-menopausal people. Few studies have also suggested its limitation in diagnosing borderline and Stage 1 tumor [25]. The IOTA-ADNEX multiclass prediction model is the first to differentiate between benign and malignant tumors, as well as to subdivide any malignancy into borderline tumors, Stage I, Stage II-IV primary malignancies, and secondary metastatic tumors [26]. A comparison of six different prediction models on 4,905 masses in 17 centers revealed that the IOTA Simple Rules risk model and IOTA-ADNEX model are better models for determining ovarian masses in females presenting with an adnexal lesion than RMI [27].

Comparison of IOTA Simple Rules and IOTA-ADNEX models

IOTA has developed the original Simple Rules (2008) to classify ovarian tumors pre-operatively. This system consists of five features for benign and malignant tumors each. Based on the features, the Simple Rules model classifies tumors into benign, malignant, and inconclusive [28]. Just like the IOTA-ADNEX model, the Simple Rules model by IOTA is widely accepted. It is a reliable tool and can act as an effective predictive tool for adnexal masses by the gynecologist. Few studies have suggested that even though we can widely accept this model in clinical settings, these approaches will be biased by the prevalence of malignant cancers as compared to benign cancer, and half of the patients with benign features have to undergo unnecessary intervention [29]. Few studies have also revealed that the use of other methods and subjective examination by an expert is needed in case of inconclusive results. The Simple Rules model offers to characterize about 75% of adnexal masses correctly also recommended using this test as triage and subjective assessments by experienced ultrasound experts for yielding better results [30].

Comparison of IOTA LR1 and LR 2 and IOTA-ADNEX models

Benign cysts require minimal invasive surgeries which in turn reduce the duration of hospitalization and early recovery than laparotomy. The IOTA group designed two LR models, LR1 and LR2. The original LR1 model has 12 variables, whereas, using a step-wise selection of variables, a simpler model (LR2) uses only the first six variables of LR1 model [31]. Many external and internal studies have suggested that the LR models can predict the outcome of the presence of ovarian malignancy in patients presenting with adnexal masses. These findings were similar to that of subjective examination by the experts. This model was externally tested and validated by experienced ultrasound sonographers [32]. As compared to LR1 and LR2 models, the IOTA-ADNEX modal can be used by non-experienced sonographers with limited years of experience. One such study was conducted in China, where they found out that their non-expert sonographers were able to distinguish between benign and malignant cancers. Hence, it is concluded to be a user-friendly, reliable, and validated tool for differentiating cancers [33]. Comparison of between various models is presented in Table 1.

**Table 1:** Comparison between various models RMI: Risk of Malignancy Index; IOTA-ADNEX: International Ovarian Tumor Analysis-assessment of different neoplasias in the adnexa; LR: Logistic Regression

Variable	RMI	IOTA-Simple Rules	IOTA-LR1 and LR2	IOTA-ADNEX
Year Developed	1990	2008	2013	2014
Use	Used for discriminating benign and malignant adnexal masses	Use to predict malignancy peri-operatively	Use to predict likelihood of malignancy	Multiclass model use to discriminate benign and malignant and also suggest stage of malignancy
System	RMI1, RMI2, RMI3, RMI4	No different scoring system	LR 1 and LR 2	Calculation is done with or without Serum CA-125 marker
Algorithm	The equation used is: the product of the menopausal status score (M), ultrasonographic score (U), and an absolute value of Serum CA-125	The Simple Rules consist of five features typical for benign tumors (B-features) and five features typical for malignant tumors (M-features)	LR1 consists of 12 selected variables. A simpler version (LR2) uses six selected variables	The IOTA-ADNEX model uses three clinical features and six ultrasound parameters with or without Serum CA-125 marker
Results Interpretation	More than 200: high risk; 25-200: intermediate risk; Less than 25: low risk	Benign: Only B-features apply; Malignant: Only M-features apply; Inconclusive: No features apply or both B- and M-features apply		Benign tumors, borderline tumors, early-stage primary cancers, late-stage primary cancers (Stages II-IV), and secondary metastatic cancers

## Conclusions

It appears that the IOTA-ADNEX model is reliable and a validated tool for predicting the adnexal masses. In this review, we discussed the various tools that were used to predict the nature of adnexal masses and classify them into benign and malignant tumors. They were subjective assessment, RMI, IOTA-Simple Rules, and IOTA-LR1 and LR2. In comparison to other pre-operative predictive models, the IOTA-ADNEX model appears to be the best fit for ruling out the malignancy and also to determine the type and stage of malignancy. This model has general acceptability and can be used by a non-expert sonographer. When executed by expert clinician and expert sonographer, it yielded the accurate results, which helped the gynecologist to plan the further course of action. This tool enhanced patient outcomes and rehabilitation by early differentiation between benign and malignant tumors. Since IOTA studied 6,000 women from 10 different developed countries, further prospective studies are need to increase the clinical knowledge and epidemiological evidences, especially for developing and underdeveloped countries.
